# Effects of Acupuncture on 1-Chloro-2,4-dinitrochlorobenzene-Induced Atopic Dermatitis

**DOI:** 10.1155/2013/982095

**Published:** 2013-08-07

**Authors:** Ji-Yeun Park, Hi-Joon Park, You Yeon Choi, Mi Hye Kim, Seung-Nam Kim, Woong Mo Yang

**Affiliations:** ^1^Studies of Translational Acupuncture Research (STAR), Acupuncture and Meridian Science Research Center (AMSRC), Kyung Hee University, 26 Kyungheedae-ro, Dongdaemun-gu, Seoul 130-701, Republic of Korea; ^2^Department of Korean Medical Science, Graduate School of Korean Medicine, Kyung Hee University, Seoul 130-701, Republic of Korea; ^3^Department of Prescriptionology, College of Korean Medicine, Kyung Hee University, 26 Kyungheedae-ro, Dongdaemun-gu, Seoul 130-701, Republic of Korea

## Abstract

Though the effects of acupuncture in atopic dermatitis have been proven in clinical studies, its mechanism remains unclear. In this study, we investigate the effectiveness and mechanism of action for acupuncture treatment on the LI11 meridian point for treatment of allergic contact dermatitis. BALB/c mice received 1-chloro-2,4-dinitrobenzene (DNCB) application to induce skin inflammation. Acupuncture treatment on LI11 significantly inhibited cutaneous hyperplasia, serum IgE levels, and expression of proinflammatory cytokine (IL-4, IL-8, and TNF-**α**) mRNA and NF-**κ**B, ERK1/2, JNK, and p38 proteins. Acupuncture treatment of local points also inhibited cutaneous hyperplasia and serum IgE levels; however, it was not effective in regulating proinflammatory cytokines and proteins. In addition, LI11 treatment is more effective at reducing serum IgE levels and pro-inflammatory cytokines and proteins than local point treatment. These results suggest that acupuncture treatment is effective in alleviating allergic contact dermatitis by reducing pro-inflammatory cytokines and proteins.

## 1. Introduction

Allergic contact dermatitis (ACD) is a chronic inflammatory skin disease presenting with cutaneous hyperreactivity that progresses due to the activation of inflammatory cells related to various allergic immune responses [1]. Although the etiology and pathology of ACD are not fully understood, previous studies suggest that typical symptoms of ACD are predominantly caused by allergen-specific T-helper (Th) 1/2 cell dysregulation, leading to immunoglobulin E (IgE) production [2, 3] and the accumulation of proinflammatory mediators [4].

The incidence of ACD has increased dramatically, especially in industrialized countries, and it now affects up to 20% of children and 3% of adults worldwide [5]. Until recently, ACD has been treated with medications such as steroid therapy and immunosuppressive agents. However, these pharmacological therapies may cause various side effects [6]. Thus, the use of less toxic alternative therapies, including acupuncture and herbal preparations, is increasing for the treatment of ACD [7–9].

Acupuncture is a nonpharmacologic technique widely used in the treatment of pain [10–12], wounds, and various skin diseases such as inflammation [13–15]. In several studies, acupuncture has been shown to reduce experimental itch, allergen-induced basophil activation, and eczema in atopic dermatitis [16–19]. Though the therapeutic efficacy of acupuncture in the treatment of atopic dermatitis has been proven in clinical studies [17, 18], its mechanism of action remains poorly understood.

In the present study, we employed a 1-chloro-2,4-dinitrobenzene- (DNCB-) induced model of ACD in mice. To evaluate the effects of acupuncture on ACD, we investigated changes in histology, total IgE serum levels, and mRNA expression of pro-inflammatory cytokines. In addition, the expression of NF-*κ*B and MAPKs (ERK1/2, JNK, and p38) was measured in the dorsal skin by western blot.

## 2. Materials and Methods

### 2.1. Animals and Treatment

BALB/c mice (7-week-old females) were purchased from Japan SLC, Inc. (Hamamatsu, Japan). Both animal care and the study protocol were conducted according to the guidelines of the Committee on Care and Use of Laboratory Animals of Kyung Hee University (KHUASP (SE)-12-020). The mice were maintained for 10 days in pathogen-free conditions before the start of the experiment. Mice were kept at a constant temperature (23°C) and humidity (55%) with a 12 h light/dark cycle, and they were provided with a laboratory diet and water *ad libitum*. After the 10-day adaptation period, mice were assigned to one of four groups (each, *n* = 5): *NOR* (normal group, mice treated with vehicle); *DNCB* ****(negative control group, mice sensitized with DNCB); *MP* ****(meridian point, mice sensitized with DNCB and treated at meridian point LI11); and *LP* (local point, mice sensitized with DNCB and treated at local points surrounding the lesion).

For induction of ACD-like skin disorders, DNCB was applied onto the mouse dorsal skin. The dorsal skin region of mice in all groups was shaved with an electric razor in preparation for each experimental cutaneous application. Induction of ACD was achieved by topical application of 100 *μ*L 1% DNCB in 4 : 1 (v/v) acetone/olive oil solution (A/O) once daily to the shaved dorsal skin. These procedures were repeated for 3 days (days 0–2) and followed by a period of no treatment for 5 days (days 3–7). In the second challenge, the LP and MP groups were treated with acupuncture 3 h prior to the application of 0.5% DNCB (days 8–16). Mice in the control group for ACD received vehicle treatment alone (A/O, 4 : 1) without DNCB treatment. Following challenge for 7 days, the mice were sacrificed on day 17 of the experiment. Skin tissues from the backs of the mice were excised and subjected to histological examination, and blood was collected in heparinized tubes from cardiac puncture. All experiments were performed blindly.

### 2.2. Histological Examination

The dorsal skin (1 × 0.5 cm) was removed and fixed in 10% paraformaldehyde (Sigma, St. Louis, MO, USA). Fixed tissues were embedded in paraffin for 24 h and serially sectioned to a thickness of 4 *μ*m for histological analysis. Tissue sections were stained with hematoxylin and eosin (H&E) and examined for general morphology. To assess epidermal and dermal hyperplasia in all four groups, all tissue samples were examined and photographed in a blinded fashion. Images were captured using the Leica Application Suite (LAS; Leica Microsystems, Buffalo Grove, IL, USA) and viewed at ×100 magnification.

### 2.3. Measurement of Total Serum IgE Levels

Blood samples were collected from the mice after sacrifice, and serum samples were obtained by centrifugation (14,000 ×g, 30 min). Total serum IgE levels of three group (*n* = 5) were measured three times repeatedly by an enzyme-linked immunosorbent assay (ELISA) kit (Cat. no. KT-401; Kamiya Biomedical, Seattle, WA, USA) following the manufacturer's instructions.

### 2.4. Detection of mRNA Expression by Reverse Transcription Polymerase Chain Reaction (RT-PCR)

To determine cytokine gene expression in the dorsal skin, reverse-transcription PCR was performed. Total RNA was extracted from the dorsal skin using TRIzol Reagent (Invitrogen Corp., Carlsbad, CA, USA) following the manufacturer's instruction and quantified by determining the OD at 260 nm. Prepared cDNAs were amplified using commercially available cDNA synthesis kits (Invitrogen Corp., Carlsbad, CA, USA) according to the manufacturer's recommendations. Thermocycler conditions consisted of an initial step at 4°C for 5 min, followed by a step at 45°C for 60 min and 40 cycles of a subsequent 2-step PCR program at 95°C for 5 min. Amplification of cDNA was conducted with Taq polymerase (Promega) and primers specific for tumor necrosis factor- (TNF-) *α*, interleukin- (IL-) 1*β*, IL-4, IL-8, and GAPDH mRNA. The primers used for amplification were synthesized using Primer Express Software (Applied Biosystems), and the sequences of the primers used in this study are shown in Table 1. The PCR cycles consisted of denaturation at 94°C for 30 s, annealing at 58°C for 30 s, and extension at 72°C for 45 s for 30 cycles. PCR products were separated by electrophoresis through a 2% agarose gel, stained with ethidium bromide, and then detected using UV light. For semiquantitative analysis of PCR bands, the density of each band was measured with a computer imaging device and accompanying software (Bio-Rad, Hercules, CA, USA).

### 2.5. Detection of Protein Expression by Western Blot Analysis

Western blotting was performed to study pro-inflammatory protein expression. Frozen skin tissues were homogenized in cytoplasmic lysis buffer (10 mM HEPES, pH 7.9, 10 mM KCl, 0.1 mM EDTA, 0.1 mM EGTA, 1 mM DTT, 0.15% Nonidet P-40, 50 mM *β*-glycerophosphate, 10 mM NaF, and 5 mM Na_3_VO_4_) containing a protease inhibitor cocktail (Roche, Indianapolis, IN, USA) and centrifuged at 500 rpm for 5 min. After removing the supernatant, the sunken pellet (nuclear pellet) was added to nuclear lysis buffer (20 mM HEPES, pH 7.9, 400 mM NaCl, 1 mM EDTA, 1 mM EGTA, 1 mM DTT, 0.50% Nonidet P-40, 50 mM *β*-glycerophosphate, 10 mM NaF, and 5 mM Na_3_VO_4_) containing protease inhibitor cocktail and then homogenized for 15 min on ice. Nuclear protein was centrifuged at 12,000 ×g for 15 min at 4°C to determine NF-*κ*B levels. MAPKs (extracellular signal-regulated kinase (ERK), Jun NH2-terminal kinase (JNK), and p38 MAPK) levels were confirmed from whole extracts. Each group of skin tissues was homogenized on ice for 15 min in RIPA buffer (50 mM Tris-HCl, pH 7.4, 1% Nonidet P-40, 0.5% sodium deoxycholate, and 150 mM NaCl) containing protease inhibitor cocktail. The resultant homogenate was centrifuged at 10,000 ×g for 30 min at 4°C, and the supernatant was collected for whole protein extraction. The protein concentration was measured using a protein assay reagent (Bio-Rad, Hercules, CA, USA), and 30 *μ*g of protein was denatured with SDS buffer. Samples were separated on a 10% SDS-polyacrylamide gel, and the proteins were then electrotransferred to a polyvinylidene fluoride (PVDF) membrane. The immunoblot was incubated overnight in blocking solution (5% skim milk) at 4°C, followed by a 4 h incubation in monoclonal anti-NF-*κ*B, ERK1/2, JNK, or p38 (Cell Signaling, CA, USA). Blots were washed two times with TBS-T and incubated with anti-rabbit alkaline phosphatase-conjugated secondary antibody (Santa Cruz Biotechnology Inc., CA, USA) for 2 h at room temperature. The proteins were then visualized using an enhanced chemiluminescence (ECL) detection reagent (Amersham Pharmacia, Piscataway, NJ, USA). The relative band density was determined using a computerized densitometry system and normalized to the *β*-actin signal from a blot developed under similar conditions.

### 2.6. Statistical Analysis

GraphPad Prism 5 software (GraphPad Software Inc., San Diego, CA, USA) was used for the statistical analysis. All data are expressed as the mean ± standard deviation (SD). Significance was determined using one-way ANOVA with the Newman-Keuls post hoc test. In all analyses, *P* < 0.05 indicated statistical significance.

## 3. Results

### 3.1. Effects of Acupuncture on Histological Changes

**Figure 1:**
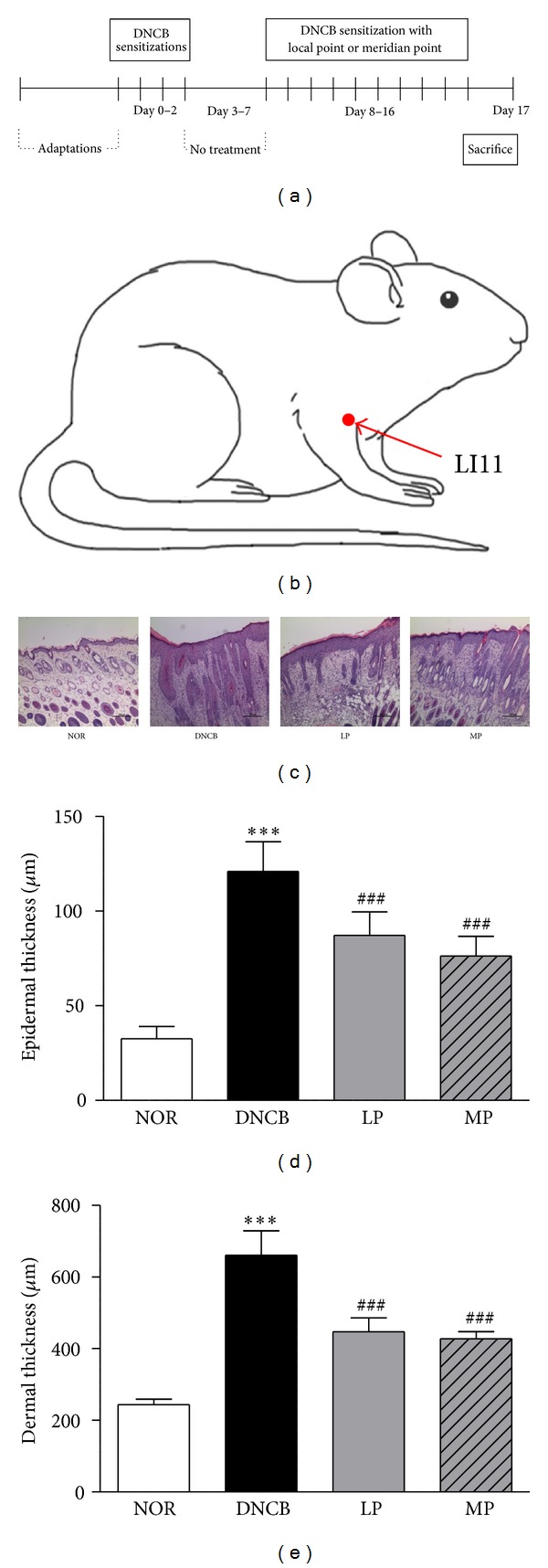
Experimental details and the result of histological analysis by H&E staining of epidermal and dermal hyperplasia. (a) Experimental schedule of DNCB sensitization and acupuncture treatment at the local and meridian points. (b) Location of the acupuncture point LI11 used in this study. LI11 is in the depression on the lateral end of the cubital crease, at the midpoint of the line connecting LU5 with the lateral epicondyle of the humerus. (c) Reduced thickening and hyperplasia after acupuncture treatment at local and meridian points in the epidermis and dermis. Scale bar: 200 *μ*m. (d-e) The skin thickness of the DNCB group was significantly increased compared to the normal group. Acupuncture treatment at the local points and meridian points significantly reduced thickening and hyperplasia in the epidermis (d) and dermis (e) compared to the DNCB group. NOR: mice treated with vehicle; DNCB: mice sensitized with DNCB; LP: mice sensitized with DNCB and treated at local points around the lesion; MP: mice sensitized with DNCB and treated at meridian point, LI11. ***P < 0.001, compared to the NOR group; ^###^ *P* < 0.001, compared to the DNCB group. One-way ANOVA followed by the Newman-Keuls test was performed for statistical analysis, and all data are presented as the mean ± S.D.

We examined whether acupuncture treatment affected the interrupted skin barrier in ACD using H&E staining. The average epidermal thickness of the dorsal skin was 32.58 ± 6.55 *μ*m in the normal group, 120 ± 15.83 *μ*m in the DNCB group, 87.16 ± 12.38 *μ*m in the LP group, and 76.30 ± 10.34 *μ*m in the MP group. Additionally, the average dermal thickness of the dorsal skin was 243.36 ± 15.37 *μ*m in the normal group, 660.97 ± 67.89 *μ*m in the DNCB group, 447.27 ± 38.55 *μ*m in the LP group, and 427.94 ± 19.89 *μ*m in the MP group. The skin was significantly thicker in DNCB-treated mice than in the normal group. Compared to the DNCB group, MP-treated mice displayed significantly reduced skin thickening and hyperplasia (*P* < 0.001). LP-treated mice also showed reduced skin thickening compared to the DNCB group (*P* < 0.001), although not to the extent of that observed in the MP group, which displayed the least amount of thickening among the groups (Figures 1(c)–1(e)).

### 3.2. Effects of Acupuncture on Serum IgE Levels

**Figure 2:**
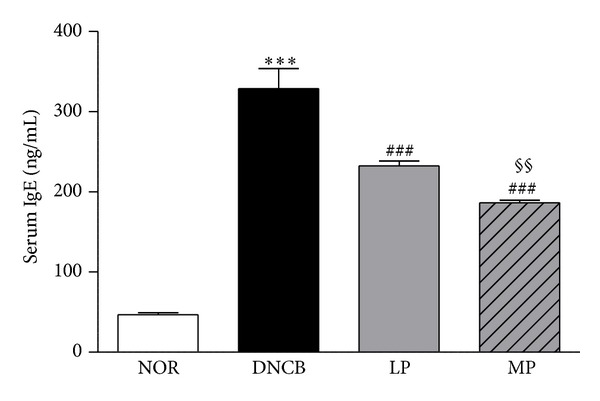
Suppression of serum IgE levels by acupuncture treatment. DNCB-mediated induction of IgE levels was significantly reduced in the MP and LP groups. IgE levels in the MP group were less than in the LP group. NOR: mice treated with vehicle; DNCB: mice sensitized with DNCB; LP: mice sensitized with DNCB and treated at local points around the lesion; MP: mice sensitized with DNCB and treated at meridian point LI11. ***P < 0.001, compared to the NOR group; ^###^ *P* < 0.001, compared to the DNCB group. ^§§^ *P* < 0.01, compared to the LP group. One-way ANOVA followed by the Newman-Keuls test was performed for statistical analysis, and all data are presented as the mean ± S.D.

Total IgE serum levels were measured to assess the effects of acupuncture treatment on DNCB-induced mice. The DNCB group showed increased IgE levels (328.87 ± 25.13 ng/mL,  *P* < 0.001) compared to the normal control group (46.95 ± 2.5 ng/mL). DNCB-mediated enhancement of serum IgE levels was significantly reduced in the MP-treated group (186.37 ± 3.23 ng/mL, *P* < 0.001) and the LP-treated group (232.47 ± 5.93 ng/mL, *P* < 0.001) compared to the DNCB group. In addition, IgE levels of the MP group were significantly reduced compared to the LP group (*P* < 0.01) (Figure 2).

### 3.3. Effects of Acupuncture on Cytokine Levels

**Figure 3:**
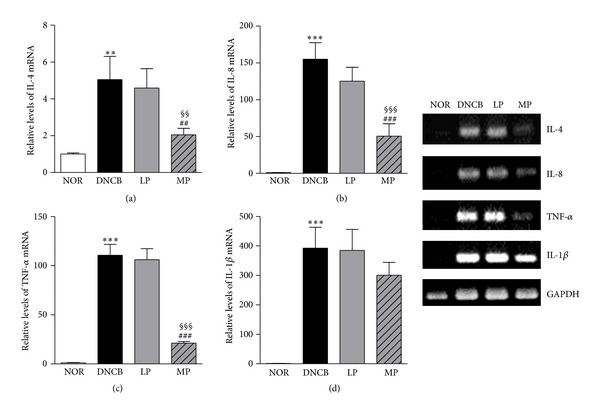
Effects of acupuncture treatment on production of cytokines. IL-4, IL-8, TNF-*α*, and IL-1*β* mRNA expression was increased significantly by DNCB sensitization. MP treatment significantly reduced IL-4, IL-8, and TNF-*α* expression. NOR: mice treated with vehicle; DNCB: mice sensitized with DNCB; LP: mice sensitized with DNCB and treated at local points around the lesion; MP: mice sensitized with DNCB and treated at meridian point LI11. **P < 0.01, ***P < 0.001, compared to the NOR group; ^##^ *P* < 0.01, ^###^ *P* < 0.001, compared to the DNCB group; ^§§^ *P* < 0.01, ^§§§^ *P* < 0.001, compared to the LP group. One-way ANOVA followed by the Newman-Keuls test was performed for statistical analysis, and all data are presented as the mean ± S.D.

RT-PCR was performed to measure the expression levels of pro-inflammatory cytokine (IL-8, TNF-*α*, and IL-1*β*) and Th2 cytokine (IL-4) mRNA in the dorsal skin. Expression of IL-4, IL-8, TNF-*α*, and IL-1*β* mRNA was induced by DNCB treatment (5.05 ± 1.25, 155.04 ± 22.49, 110.67 ± 11.16, and 392.82 ± 70.59, resp.). IL-4, IL-8, and TNF-*α* mRNA expression was drastically downregulated in the dorsal skin of MP-treated mice (2.05 ± 0.35, 50.58 ± 16.87, and 21.21 ± 1.61, resp.) but not in the LP group (Figure 3). In addition, the level of IL-4, IL-8, and TNF-*α* mRNA was significantly reduced in the MP group compared to the LP group. Expression of IL-1*β* mRNA followed an expression pattern similar to that of the other cytokines in the treatment groups, but it was not statistically significant.

### 3.4. Effects of Acupuncture on NF-*κ*B Expression in the Skin

**Figure 4:**
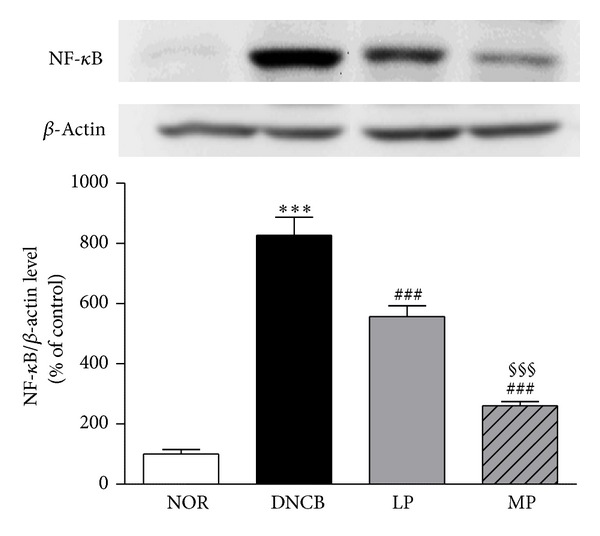
Suppression of NF-*κ*B expression by acupuncture treatment. Increased expression of NF-*κ*B was significantly reduced upon MP and LP treatment. The MP group showed reduced expression compared to the LP group. NOR: mice treated with vehicle; DNCB: mice sensitized with DNCB; LP: mice sensitized with DNCB and treated at local points around the lesion; MP: mice sensitized with DNCB and treated at meridian point LI11. ***P < 0.001, compared to the NOR group; ^###^ *P* < 0.001, compared to the DNCB group; ^§§§^ *P* < 0.001, compared to the LP group. One-way ANOVA followed by the Newman-Keuls test was performed for statistical analysis, and all data are presented as the mean ± S.D.

To investigate the anti-inflammatory mechanism of acupuncture treatment in ACD-like disorders, the effects of acupuncture on activation of NF-*κ*B, an important transcription factor that mediates the transcription of many pro-inflammatory cytokine genes, were examined by western blotting. MP and LP treatment significantly reduced the expression of NF-*κ*B (MP: 260.02 ± 14.67, *P* < 0.001; LP: 556.87 ± 36.24, *P* < 0.001) compared to the DNCB group (826.82 ± 60.63). In addition, the MP group displayed greater anti-inflammatory efficacy than the LP group (*P* < 0.001)**  **(Figure 4).

### 3.5. Effects of Acupuncture on Expression of MAPKs in the Skin 

**Figure 5:**
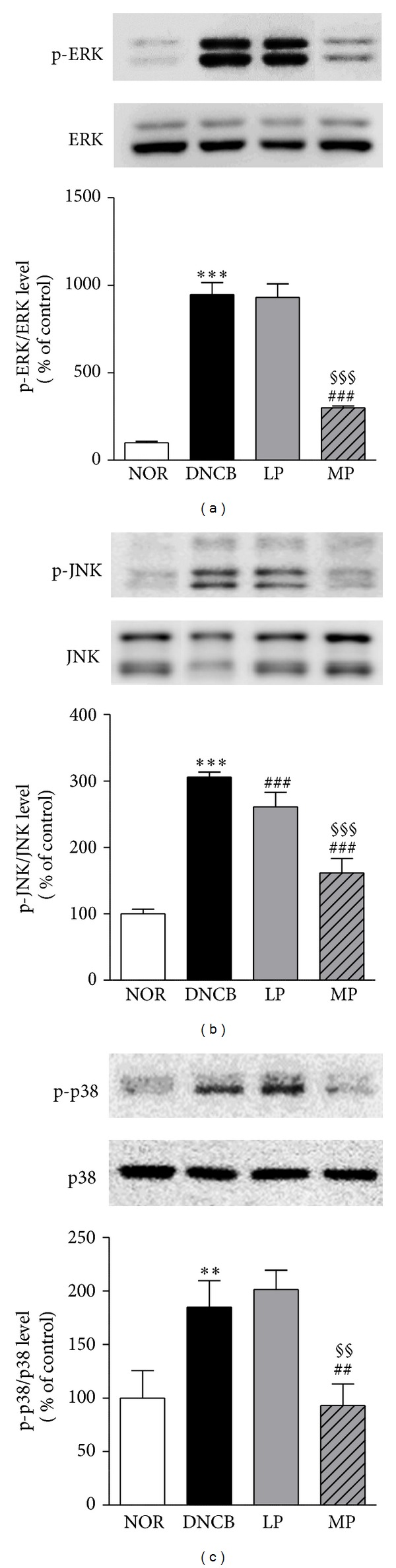
Suppression of MAP kinase expression by acupuncture at the meridian point LI11. Phosphorylation of ERK1/2, JNK, and p38 MAP kinases was significantly elevated by DNCB sensitization. Acupuncture treatment on the meridian point significantly suppressed the expression of MAP kinases. NOR: mice treated with vehicle; DNCB: mice sensitized with DNCB; LP: mice sensitized with DNCB and treated at local points around the lesion; MP: mice sensitized with DNCB and treated at meridian point LI11. **P < 0.01, ***P < 0.001, compared to the NOR group; ^##^ *P* < 0.01, ^###^ *P* < 0.001, compared to the DNCB group; ^§§^ *P* < 0.01, ^§§§^ *P* < 0.001, compared to the LP group. One-way ANOVA followed by the Newman-Keuls test was performed for statistical analysis, and all data are presented as the mean ± S.D.

The DNCB group displayed increased expression of MAPK signaling proteins: p-ERK1/2, p-JNK, and p-p38 in the MAPK signaling pathway (946.89 ± 68.80, 305.89 ± 7.64, and 184.81 ± 24.64, resp.). MP treatment inhibited the upregulation of phosphorylation of ERK1/2, JNK, and p38 (299.95 ± 10.39, 161.66 ± 21.35, and 93.00 ± 20.04) compared with the DNCB group. In addition, the levels of p-ERK1/2 and p-38 in the LP group were not statistically different from, the DNCB group, whereas the change in expression of p-JNK was statistically significant. MP treatment reduced the levels of p-ERK1/2, p-JNK, and p-p38 more effectively than LP treatment (930.88 ± 77.37, 260.99 ± 21.77, and 201.35 ± 18.08, resp.) (Figure 5).

## 4. Discussion

Although acupuncture treatment has been increasingly used in atopic dermatitis, with several clinical studies demonstrating the effectiveness of acupuncture [17, 18], preclinical studies, especially in vivo studies, investigating the mechanism of action for acupuncture are lacking. To the best of our knowledge, this is the first study to identify acupuncture-mediated anti-inflammatory mechanisms in atopic dermatitis.

Several methods for selecting acupuncture points to treat dermatitis exist. One method involves selection of meridian points at locations remote from the lesion, such as LI11 and LI4 [20, 21]. An alternative method selects local points around the lesion [14]. Among the meridian points, LI11 is known to have therapeutic effects on various skin diseases such as inflammation, pruritus, and urticaria [20–22]. In this study, we investigated the therapeutic effect of the LI11 meridian point compared to local points around the lesion to define potential differences of the effect based on the location of acupuncture treatment.

ACD is defined as inflammatory skin disease characterized by pruritic and eczematous skin lesions, extensive inflammatory cell infiltration, and release of pro-inflammatory mediators [23]. To evaluate the histological changes after MP treatment in DNCB-induced mice, dorsal skin sections were subjected to H&E staining. Hypertrophy and hyperkeratosis of the dermis and epidermis were observed in our DNCB-induced model. In contrast, the MP and LP groups displayed significant reductions in DNCB-induced hyperplasia of epidermal and dermal thickening. Though there was no significant difference between the MP and LP groups, the reduction of DNCB-induced hyperplasia and epidermal thickening was a little more pronounced in the MP group.

IgE expression was known to cause both acute and chronic phase skin inflammations. Therefore, the upregulation of total serum IgE is a hallmark of ACD [24]. In the present study, the concentration of total serum IgE was reduced in both MP- and LP-treated mice as compared to the DNCB group. Interestingly, meridian point acupuncture treatment is more effective in reducing allergic sensitization and the severity of ACD than local acupuncture treatment.

We next examined the effect of acupuncture treatment on cytokine responses. In the pathogenesis and progression of ACD, the regulation of inflammatory cytokine production is an essential step. Important roles of Th1 and Th2 cytokines have been confirmed in DNCB-induced allergic skin inflammation models by targeting deletions of these cytokines [25]. Generally, contact allergens have been associated with increases in the Th1 cytokines, INF-*γ* and TNF-*α*. In particular, TNF-*α* is an essential mediator of ACD [26]. TNF-*α* expression is increased after challenge with DNCB and only showed a significant reduction in MP treatment. Also, we showed that MP treatment decreased Th2 cytokine IL-4, which plays a central role in the promotion of an allergic inflammatory eosinophilic reaction in allergic diseases through IgE isotype switching [25]. Therefore, our results suggest that MP treatment can reduce serum IgE by suppressing the Th1 response as well as Th2. In addition, IL-8, a member of the chemokine family, is produced by various types of cells upon stimulation with inflammatory stimuli [27]. A pathological increase of cutaneous IL-1*β* is associated with edema formation, epidermal hyperproliferation, and contact atopic dermatitis in humans [27]. Our data showed that IL-8 and IL-1*β* expression is increased after challenge with DNCB and only showed a significant reduction of IL-8 reduction in MP treatment. Meanwhile, LP treatment did not affect any of the expression of pro-inflammatory cytokines. These results showed that acupuncture treatment on the LI11 meridian point is more effective in regulating pro-inflammatory, Th1 and Th2, cytokines than local point treatment.

Transcription factors of the NF-*κ*B family have also been implicated in the arrest of proliferation and initiation of differentiation in epidermal keratinocytes [28]. Additionally, many studies have reported that the MAPK signaling cascade plays an essential role in the initiation of inflammatory responses [29]. In addition, activation of the MAPK signaling pathway ultimately results in direct or indirect phosphorylation and/or activation of NF-*κ*B, as well as alterations in gene expression [30]. These findings indicate that the MAPK pathway could be an effective target for anti-inflammatory therapy. We confirmed that increased expression of NF-*κ*B and MAPKs by DNCB treatment was subsequently reduced after MP treatment. The results suggest that MP treatment has anti-inflammatory effects by inhibiting pro-inflammatory activities. This anti-inflammatory effect of MP treatment might be produced through the systemic immune modulations which are connected to central nerve changes induced by acupuncture treatment [31–37]. We also found that LP treatment influenced regulating NF-*κ*B and p-JNK. It might be mainly produced by local anti-inflammatory action of acupuncture stimulation [14]. Overall, effect of LP treatment was less powerful than MP treatment and it did not affect pro-inflammatory cytokines and proteins. From these results, we suggest that MP treatment is more effective in regulating pro-inflammatory biomarkers speculated to be exerted by systemic modulation.

In summary, we demonstrated that acupuncture treatment on the LI11 meridian point is an effective means to reduce mouse dorsal skin hyperplasia and serum IgE levels in ACD, and these effects were mediated by regulating pro-inflammatory cytokines (IL-4, IL-8, and TNF-*α*) and proteins (NF-*κ*B and MAPKs). In contrast, local point treatment was also effective in reducing dorsal skin thickness and serum IgE levels; however, it did not affect most of pro-inflammatory cytokines and proteins, which indicates that the therapeutic effect of local stimulation in ACD might be produced by pathways other than pro-inflammatory pathways. These results demonstrate that acupuncture may be a useful treatment for the treatment of ACD. Further investigation would be necessary to clarify its molecular mechanisms of action.

## Figures and Tables

**Table 1:** Primer sequences.

Target gene	Primer	Sequence (5′-3′)	Amplicon size (bp)
IL-8	F	5′-TGTGGGAGGCTGTGTTTGTA-3′	151
	R	5′-ACGAGACCAGGAGAAACAGG-3′	

IL-4	F	5′-TCATCGGCATTTTGAACGAG-3′	399
	R	5′-CCCATACTTTAGGAAGACACGGATT-3′;	

IL-1*β*	F	5′-CTC TAG ACC ATG CTA CAG AC-3′	291
	R	5′-TGG AAT CCA GGG GAA ACA CTG-3′	

TNF-*α*	F	5′-GGT GCA ATG CAG AGC CTT CC-3′	173
	R	5′-CAG TGA TGT AGC GAC AGC CTG G-3′	

GAPDH	F	5′-GGC ATG GAC TGT GGT CAT GA-3′	376
	R	5′-TTC ACC ACC ATG GAG AAG GC-3′	
